# Prevalence of bacteria involved in bovine respiratory disease in dairy heifers in Spain: influence of environmental factors

**DOI:** 10.3389/fvets.2025.1605045

**Published:** 2025-06-30

**Authors:** Sandra Barroso-Arévalo, Michela Re, José María San Miguel Ayanz, Eugenia Peralta Val, Alberto Alvarado-Piqueras, Rocío Fernández-Valeriano, Javier Blanco-Murcia

**Affiliations:** ^1^Department of Internal Medicine and Animal Surgery, Faculty of Veterinary Medicine, Complutense University of Madrid, Madrid, Spain; ^2^Zoetis Spain S.L.U., Madrid, Spain; ^3^Group of Rehabilitation of the Autochthonous Fauna and Their Habitat (GREFA), Wildlife Hospital, Madrid, Spain

**Keywords:** bovine respiratory disease, bacterial prevalence, dairy heifers, *Mycoplasma bovis*, *Trueperella pyogenes*, risk factor, cattle disease management

## Abstract

**Introduction:**

Bovine Respiratory Disease (BRD) is a multifactorial condition and a major health and economic concern in dairy production.

**Methods:**

This study aimed to determine the prevalence of five key bacterial pathogens—*Mannheimia haemolytica*, *Pasteurella multocida*, *Trueperella pyogenes*, *Mycoplasma bovis*, and *Histophilus somni*—in Spanish dairy heifers and to evaluate the influence of seasonality, geographical location, farm size, and antibiotic use. In 2017, samples (deep nasopharyngeal swabs, transtracheal aspirates, and blood) were collected from 855 heifers (<12 months old) in 50 farms across Spain. Bacterial isolation and serological testing (ELISA) were performed.

**Results:**

*Mycoplasma* spp. showed the highest overall prevalence (26.7% at the individual level by culture; 75.7% of cultured farms), with PCR confirming *M. bovis* in 89% of Mycoplasma-positive farms. Serology revealed 16.3% individual-level positivity for *M. bovis* and 63% farm-level positivity. *T. pyogenes* was also notable, detected in 6.0% of animals (45% of farms). Lower isolation rates were observed for *M. haemolytica* (2.5%) and *P. multocida* (3.5%), while *H. somni* was not detected. Larger farm size, winter season, and certain regions were significantly associated with higher prevalence of *M. bovis* and *T. pyogenes*.

**Discussion:**

These findings underscore the need for improved management practices—such as better ventilation, reduced stocking density, and targeted vaccination—to mitigate BRD risk in high-prevalence settings.

## 1 Introduction

Bovine Respiratory Disease (BRD) is one of the most significant respiratory diseases affecting cattle worldwide, particularly in intensive production systems. It is a leading cause of morbidity and mortality in calves, with major implications for animal health, welfare, and productivity during early development (1–3). The economic impact of BRD is substantial, encompassing both direct and indirect costs (4). Direct costs of BRD include veterinary treatment, mortality, pharmaceuticals, and labor, while indirect costs stem from reduced growth rates, impaired feed efficiency, and decreased milk production. These economic impacts are particularly pronounced in dairy operations, where optimal heifer growth is crucial for future milk yield and reproductive performance (5, 6). Furthermore, broader studies emphasize the often underestimated burden of BRD, highlighting its association with increased mortality and long-term effects on productivity, including delayed reproductive milestones and reduced milk production in affected heifers (7–10). For instance, a recent dataset of 104,100 dairy replacement heifers across the USA revealed that 36.6% had one or more cases of BRD diagnosed within the first 120 days of life, with the highest risk occurring before weaning. In regions such as California, BRD has been reported to affect up to 26% of dairy heifers (11). In Spain, BRD prevalence is notably high in dairy systems, exacerbating its economic and health impacts within the livestock industry (12).

BRD is characterized by a multifactorial etiology that involves a combination of bacterial and viral pathogens, environmental stressors, host immunity, and management practices. The interactions between these factors complicate both the prevention and treatment of BRD. Pathogens such as bacteria and viruses can work synergistically to overwhelm the host’s immune system, resulting in severe respiratory illness (5, 13). Environmental conditions such as overcrowding, poor ventilation, and stress due to transport or handling further contribute to the risk of disease by weakening the animal’s immune defenses (14, 15).

The bacterial pathogens most implicated in BRD include *Mannheimia haemolytica* (MH), *Pasteurella multocida* (PM), *Histophilus somni* (HS), and *Mycoplasma bovis* (MYC). These bacteria are often found in the upper respiratory tract of clinically healthy calves, existing as opportunistic pathogens that can cause disease when the animal is stressed or immunocompromised (5, 16, 17). *Mannheimia haemolytica* is particularly known for its production of leukotoxin, which contributes to lung tissue damage (18), while *Mycoplasma bovis* is associated with chronic respiratory disease and can present therapeutic challenges due to its intrinsic reduced susceptibility to various antimicrobial classes. Indeed, multiple studies have reported strains exhibiting resistance to antimicrobials such as fluoroquinolones, macrolides, and tetracyclines; however, standardized antimicrobial susceptibility breakpoints have not yet been established by either CLSI or EUCAST for this pathogen (19). Notably, *Mycoplasma bovis* has emerged as a key pathogen in Spanish dairy herds (20).

The multifactorial nature of BRD complicates its prevention and management, particularly in intensive dairy systems where stress factors such as overcrowding, poor ventilation, and seasonal fluctuations play a major role. While advancements in diagnostic tools, such as ultrasonography and PCR, have improved pathogen detection, the integration of these tools into routine management remains limited (21). Subclinical cases, where animals harbor pathogens without overt clinical signs, further hinder timely interventions and contribute to pathogen spread within herds (2, 22).

Regional studies report variability in BRD morbidity rates, ranging from 23 to 60%, depending on diagnostic methods and management practices (15, 22, 23). These rates can be even higher when using combined diagnostic approaches, such as clinical evaluation and lung ultrasonography, with prevalence estimates exceeding 60% in some populations (21). This variability in disease prevalence underscores the importance of accurate and timely diagnosis to implement appropriate control measures. Overreliance on antibiotics may not only fail to ensure full recovery but also promote resistant bacterial strains, complicating treatment option (7, 24, 25). Thus, judicious antibiotic use, combined with preventive measures such as vaccination and improved management practices, is essential for effective BRD control (24).

Despite advances in understanding the multifactorial nature of BRD and its significant economic and health impacts, critical knowledge gaps remain regarding the interplay of regional, environmental, and management factors that shape pathogen prevalence and disease outcomes. The variability observed in pathogen distribution across geographic regions and production systems underscores the necessity for localized studies that address these complexities. In Spain, where intensive dairy operations are a cornerstone of agricultural production, a comprehensive evaluation of bacterial prevalence and its associated risk factors is essential. This study aims to determine the prevalence of bacterial pathogens associated with BRD in Spanish dairy farms and evaluate the influence of factors such as seasonality, farm size, geographical region, and antibiotic use. By identifying these drivers, we hope to inform targeted management and control strategies to mitigate the burden of BRD and improve herd health and productivity.

## 2 Materials and methods

### 2.1 Study design

The study was conducted throughout 2017, from January 23 to December 2, in 50 dairy cattle farms across Spain. Farms were selected to ensure representation of different geographical regions, farm sizes, and management practices to minimize selection bias. These farms represented a total census of 33,250 heifers, with 16,000 of them being under 12 months of age. According to the latest national data, the total heifer population in Spain was 274,980 in 2022, placing the farms in this study represented approximately 12% of the national census (26). Samples were collected from 855 replacement heifers under 12 months of age, housed in separate pens on straw bedding.

Farms were categorized as small, medium, or large based on the total number of heifers present at the time of sampling. Thresholds were defined using quartile distribution across the full dataset of 50 farms: Small farms: fewer than 100 heifers; Medium farms: 100–250 heifers; Large farms: more than 250 heifers. These categories were chosen to reflect the natural distribution of herd sizes observed in the study and allow for meaningful statistical comparison. There is currently no official classification of dairy heifer farm sizes at the national level in Spain. Farms were not selected from an official national livestock database. Instead, selection was based on voluntary participation among farms distributed across the main cattle-producing regions of Spain. Regional stratification was applied to ensure representation from different climatic and management systems.

### 2.2 Sample collection

Samples for serological testing were collected from at least 7–12 animals per group in heifer lots where sick animals were observed. These samples were taken 30–40 days after a stressful handling activity, such as regrouping or transportation. Blood samples were drawn from the coccygeal vein using Vacutainer tubes and were transported in a refrigerated container within 24 h to a specialized laboratory (EuroFins, Barcelona). Following centrifugation, the serum was analyzed using an indirect ELISA technique (Bio-XÒ, Elisa Kit, Brussels, Belgium) to determine the presence of antibodies against *Mycoplasma bovis*. Serological testing was conducted exclusively for *Mycoplasma bovis* due to the recognized importance of *M. bovis* as a major contributor to chronic respiratory infections in cattle, which often result in persistent herd-level impacts and require long-term management. In addition, resource constraints limit the ability to extend serological testing to other BRD-associated pathogens. It should be noted that serological samples were obtained from a separate set of animals, and antimicrobial treatment status was not recorded for this group. Consequently, no analysis could be performed to assess the relationship between antibody prevalence and prior antibiotic exposure.

Samples for bacterial culture and identification were collected only from sick animals or animals in contact with sick animals that did not receive antibiotic treatment within the previous seven days. Samples were taken using two methods: deep nasopharyngeal swabs (DNS) and transtracheal aspirations (TTW). Sterile swabs (Culture Swab Double Guarded 33″ Jorgenssen Laboratories, Loveland, Colorado, United States) were inserted through the nasal passage to reach the nasopharynx, and rotated multiple times to collect sufficient material. The double-guarded plastic sheath was used to avoid contamination with normal nasal flora. For TTW collection, the tracheal area was shaved, disinfected with alcohol, and punctured using a 14 Gauge catheter (Vygon^®^ CentraCath, Ecouen, France). After the puncture, 20 mL of sterile saline solution (Braun, Barcelona, Spain) was injected and then aspirated using a 20- or 50-mL syringe. Samples were transported under refrigeration within 24 h to a Laboratorio Central de Veterinaria (Algete, Spain), where they were cultured on Columbia blood agar plates (Thermo Fisher Scientific Inc., Waltham, Massachusetts, United States). for *Mannheimia haemolytica*, *Pasteurella multocida*, *Trueperella pyogenes*, and *Histophilus somni* isolation and on PPLO agar plates (Thermo Fisher Scientific Inc., Waltham, Massachusetts, United States). to isolate *Mycoplasma* spp. According to established protocols for BRD studies, a farm is considered to be infected if at least there is one positive animal.

### 2.3 PCR for *Mycoplasma* identification

To determine the specific prevalence of *Mycoplasma bovis* and to rule out false positives from other *Mycoplasma* species, a pooled sample of all positive cultures from each farm was analyzed using PCR. Colonies suspected to be compatible with *Mycoplasma* spp. were collected in cryovials containing a preservation solution composed of water, glycerol, and FBP (Thermo Fisher Scientific Inc., Waltham, Massachusetts, United States). These samples were stored at −80°C until further processing. For DNA extraction, 200 μL of the thawed cell culture medium was used. The lysis process involved boiling the samples at 120°C for 10 min on a Grant UBD4^®^ heating plate (Grant Instruments^™^, Cambridge, UK), ensuring effective cell membrane disruption to release the DNA.

The extracted DNA was subjected to quantitative PCR (qPCR) using a QuantStudio 3 Real-Time PCR System^®^ (Applied Biosystems, Foster City, CA, United States). Each 20 μL qPCR assay included 15 μL of MycBov dtec-qPCR Master Mix^®^ (genetic PCR solutions^™^) and 5 μL of extracted template DNA. The cycling conditions for the qPCR were set according to the protocol provided by genetic PCR solutions^™^, with an initial activation step at 95°C for 2 min, followed by 40 cycles of denaturation at 95°C for 5 s, and hybridization and extension at 60°C for 20 s. Negative and positive amplification controls were included in each reaction to validate the absence or presence of contamination and ensure the effectiveness of the PCR reagents.

The resulting qPCR data were analyzed using QuantStudio software^®^ (Applied Biosystems). The software facilitated the interpretation of amplification curves and the determination of cycle threshold (Ct) values. Samples with Ct values greater than 40 were classified as negative for *Mycoplasma bovis*, indicating that bacterial DNA was either absent or below the detection limit of the assay.

### 2.4 Climatic characteristics of the study area

The study was conducted across four major geographical regions of Spain: Central, Eastern, Northern, and Southern areas. These regions are characterized by distinct climatic profiles. Spain’s climate during the study period is characterized by regional and seasonal variability. The country generally experiences a Mediterranean climate with hot, dry summers and mild, wetted winters, though northern areas show more oceanic influences. According to the Spanish Meteorological Agency (AEMET), annual precipitation varies significantly across regions: the northern zone (e.g., Galicia, Cantabria, Basque Country) was classified as very humid in 2019, with precipitation frequently exceeding 1,200 mm. In contrast, central and southern areas showed more variable or dry patterns, with reduced rainfall during key months. The eastern and southeastern regions experienced intense rainfall events in autumn, contributing to higher annual precipitation. Average temperatures across Spain in 2019 reached 15.9°C, exceeding the historical mean by +0.8°C, with summer and autumn being notably warm (48). These climatic differences likely influenced the survival and transmission of respiratory pathogens in cattle and were considered in the interpretation of seasonal and regional prevalence patterns.

### 2.5 Statistical analysis

The study aimed to determine two prevalence values for each microorganism (farm-level and individual-level). Farm-level prevalence was defined as the percentage of farms testing positive for each microorganism out of the total farms sampled (50 for serology and 37 for culture). Individual prevalence was defined as the number of animals testing positive in relation to the total number of samples collected (540 for serology and 315 for bacterial culture). Data are presented as mean ± SD. The association between different factors (geographical region, season, farm size, and antibiotic treatment) and bacterial prevalence was evaluated using the chi-square test. A *p*-value < 0.05 was considered indicative of a significant correlation between variables. Statistical analysis was performed using SAS^®^ software (version 9.4).

## 3 Results

### 3.1 Sample distribution

A total of 855 samples were obtained for serological (540 sera) and bacteriological analysis [315 deep nasopharyngeal swabs (DNS) or transtracheal aspirations (TTW)]. These were stratified by geographical region, farm size, treatment status, and season, providing comprehensive insights into the prevalence and distribution of bovine respiratory pathogens. The detailed distribution is presented in Table 1.

**Table 1:** Distribution of analyzed samples according to geographical region, farm size, antibiotic treatment status, and season.

Parameter	Samples for culture (*n*)	Samples for serology (*n*)	Total samples (*n*)	Number of farms
Geographical region				
Central	28	81	109	4
East	116	160	276	12
North	107	210	317	28
South	64	89	153	6
Farm size				
Small	45	145	190	25
Medium	148	197	345	10
Large	122	198	320	15
Treatment status				
No antibiotics	216	-	216	
Antibiotics	99	-	99	
Season				
Winter	141	214	355	
Autumn	143	209	352	
Spring/summer	31	117	148	
Total	315	540	855	

Each parameter (region, farm size, treatment status, and season) represents different classification criteria applied to the same set of samples. Therefore, the totals presented in each parameter category are not cumulative across parameters but rather reflect different groupings of the same 855 animals analyzed in this study. The overall total of samples collected was *n* = 855, with 315 samples analyzed by bacterial culture and 540 samples analyzed by serology.

### 3.2 Serological analysis for *Mycoplasma bovis*

From the 540 serological samples analyzed, 81 animals tested positive for *Mycoplasma bovis.* This corresponds to an individual prevalence of 15% and a farm-level prevalence of 63%.

### 3.3 Bacteriological culture results

**Figure 1:**
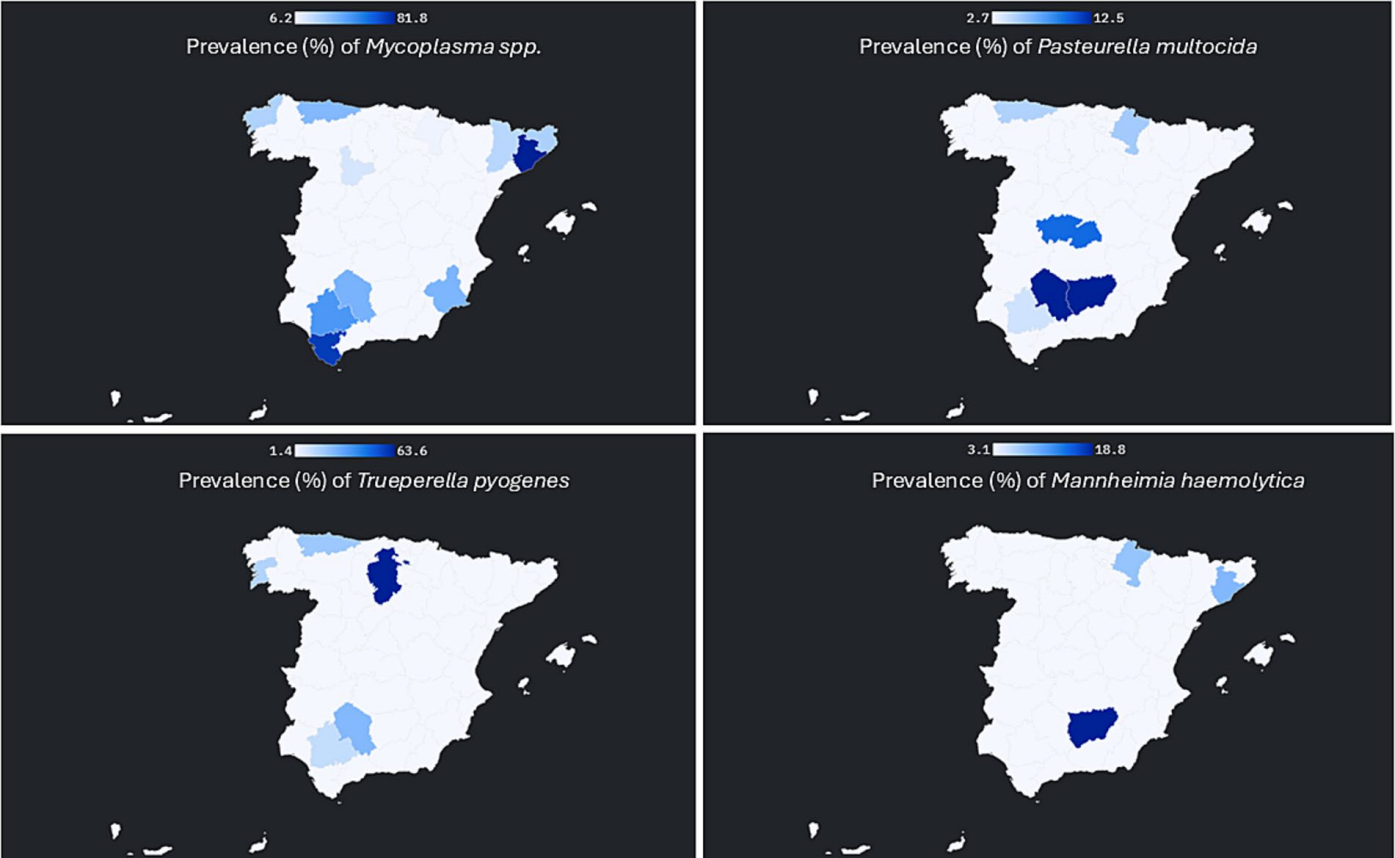
Provincial distribution of four bovine respiratory pathogens in Spain (September 2015 – March 2017). Spanish provinces are shaded by the percentage of sampled cattle that tested positive; darker tones indicate higher prevalence (see legend). Only provinces with at least one positive animal are shown. Pathogens included: *Mannheimia haemolytica, Pasteurella multocida, Arcanobacterium Trueperella pyogenes* and *Mycoplasma* spp. *Histophilus somni* was not detected and is therefore not represented.

Samples were obtained from 37 out of the 50 selected farms using DNS and/or TTW. The analysis revealed the following prevalence rates (Table 2). Spatial distribution of the positive samples for bacteriological culture are also showed in Figure 1.

**Table 2:** Number of positive samples, individual prevalence and farm-level prevalence.

Pathogen	Positive samples for culture	Individual prevalence	Farm-level prevalence	95% confidence interval
*Mannheimia haemolytica* (MH)	8/315	2.5%	21%	1.1–4.9
*Pasteurella multocida* (PM)	11/315	3.5%	16%	0.4–3.2
*Trueperella pyogenes* (TP)	19/315	6.0%	45%	3.7–9.2
Mycoplasma spp.	84/315	26.7%	75.67%	12.7–21.1
*Histophilus somni* (HS)	0/315	-	-	0.0–1.2

### 3.4 PCR analysis for *Mycoplasma bovis*

To determine the specific prevalence of *Mycoplasma bovis* and rule out false positives from other *Mycoplasma* spp., a pooled sample of all positive cultures from each farm was analyzed using PCR. Of the 28 farms that initially tested positive for *Mycoplasma* spp. through culture, the presence of *Mycoplasma bovis* was confirmed in 25 farms (89.28%), resulting in a farm-level prevalence of 67.56%.

### 3.5 Factors influencing bacterial prevalence

Several factors were found to significantly influence the prevalence of bacterial agents. For *Mycoplasma* spp., larger farms exhibited a notably higher prevalence (*p* = 0.0029), with the central region showing the highest prevalence rates (*p* < 0.0001). Seasonality also played a critical role, with winter demonstrating a marked increase in prevalence compared to other seasons (*p* < 0.0001).

*Trueperella pyogenes* showed the highest prevalence in medium-sized farms (*p* = 0.0114), while *Mannheimia haemolytica* was notably absent in small farms (*p* = 0.0219). No isolations of *Histophilus somni* were detected across any farms. *P. multocida* did not show any significant correlation with any of the studied factors (see Table 3).

**Table 3:** This table summarizes statistically significant associations (*p* < 0.05) between categorical factors and pathogen prevalence in dairy heifers.

Pathogen	Associated variable	*p*-value	Higher prevalence in
Mycoplasma spp.	Herd size	0.0029	Large farms (27.9%)
Mycoplasma spp.	Region	<0.0001	Central región (40.9%)
Mycoplasma spp.	Season	<0.0001	Winter (36.7%)
*T. pyogenes*	Herd size	0.0114	Medium herds (14.58%)
*T. pyogenes*	Season	<0.0001	Winter (12.8%)
*M. haemolytica*	Herd size	0.0219	Medium (6.3%), Large (4.1%)

### 3.6 Inter-bacterial associations

A significant association was observed between *Trueperella pyogenes* and *Mycoplasma* spp. (*p* < 0.0001), with coinfection detected in 27 samples (8.6% of the total), and a statistically significant relationship (Chi-square test, *p* = 0.0001).

## 4 Discussion

This study provides comprehensive insights into the prevalence and distribution of key bacterial pathogens associated with BRD in dairy heifers. Among the analyzed pathogens, *Mycoplasma bovis* exhibited the highest individual prevalence (16.3%), followed by *Trueperella pyogenes* (6.0%) and *Mannheimia haemolytica* (2.5%). Notably, no isolates of *Histophilus somni* were detected. However, this absence may also highlight limitations in diagnostic approaches or the influence of specific regional factors in Spain, as *H. somni* is well-documented in other geographic regions with distinct climatic and management conditions (27). These results highlight the importance of regionally adapted diagnostic strategies.

The study also identified significant factors influencing pathogen prevalence, including farm size, seasonality, and geographical region. In our study, larger farms exhibited significantly higher prevalence rates for *Mycoplasma bovis* (*p* = 0.0029), potentially due to increased animal density and stress levels, which can facilitate pathogen transmission. This aligns with findings from recent studies (28), in which the authors demonstrated that high-density systems amplify contact rates between animals, increasing the likelihood of disease outbreaks. The use of PCR for *Mycoplasma bovis* provided additional specificity, confirming its presence in 89.28% of farms that tested positive via culture. This high confirmation rate highlights the diagnostic advantages of molecular tools in differentiating *M. bovis* from other *Mycoplasma* spp., ensuring accurate prevalence estimates.

Additionally, frequent mixing of animals and broad antimicrobial use in larger farms may destabilize the microbial ecosystem, creating favorable conditions for opportunistic pathogens like *T. pyogenes* and *M. haemolytica* (29). A recent study by Centeno-Martinez et al. (30) found that cattle with BRD had significantly lower alpha diversity in their nasal microbiota compared to healthy animals, suggesting a loss of microbial stability. This microbial dysbiosis could increase susceptibility to opportunistic infections, as a less diverse microbiome may lack protective commensal interactions (31). Healthy cattle exhibited more robust bacterial co-occurrence networks, while BRD-affected animals showed fewer microbial associations, suggesting that microbiome stability plays a key role in disease resistance. Additionally, *T. pyogenes* has been strongly associated with poor management practices, such as oversized teats, forced suckling, and inadequate colostrum intake, which may explain its notable presence in certain farms included in this study.

In contrast, smaller farms demonstrated lower prevalence rates, likely due to reduced animal density and less frequent animal turnover, which limit opportunities for pathogen transmission. Extensive systems, which are characteristic of smaller farms, have been shown to reduce BRD incidence by minimizing stress and pathogen exposure (15). However, these systems might also have less robust biosecurity measures, potentially posing risks under specific circumstances. For instance, factors such as mixing animals of different sources or ages, poor biosecurity protocols, and insufficient vaccination strategies have been highlighted as contributors to respiratory disease outbreaks, even in extensive settings (32). Furthermore, inadequate surveillance and delays in identifying subclinical infections are often more pronounced in smaller systems, which can result in missed opportunities for early intervention (33). While extensive systems typically reduce the overall pathogen load through lower stocking densities, the absence of formal monitoring tools, such as scoring systems or ultrasound diagnostics, could mask disease burdens until clinical signs appear. However, when contextualized regionally, intensive management systems in central Spain may serve as hotspots for pathogen dissemination, particularly during winter when environmental stressors are compounded.

Seasonality also plays a critical role in the prevalence and distribution of bovine respiratory pathogens, as highlighted in this study. The significant increase in prevalence during winter corroborates findings from other regions, where colder months are associated with higher rates of respiratory diseases in cattle (34–36). During colder months, indoor housing often results in poor ventilation and overcrowding, facilitating the spread of airborne pathogens. Furthermore, climatic factors, such as sharp temperature variations between seasons, may exacerbate winter-associated risks, as noted in other studies from Southern Europe (14, 37). In addition, the need to shelter animals during colder months often leads to overcrowding and diminished air quality, which, in turn, facilitates the transmission of airborne pathogens. The role of air quality and ventilation in modulating the microbial load in dairy farms has been reported, suggesting that poor ventilation in winter exacerbates pathogen survival and spread (38). In Spain, meteorological data from the Spanish Meteorological Agency (AEMET) indicate that winter months are characterized by frequent cold-air outbreaks, significant diurnal temperature oscillations, and increased thermal amplitude compared to the rest of the year, particularly in continental regions. These climatic patterns help contextualize the higher BRD prevalence observed during winter in our study, particularly in areas such as central Spain, where abrupt shifts between daytime warmth and nighttime cold may place additional stress on housed animals. Furthermore, Pardon and Buczinski (22) noted peaks in *M. bovis* and *M. haemolytica* prevalence during colder months, supporting the findings of this study. Interestingly, while our study did not find significant prevalence shifts for *Pasteurella multocida* across seasons, literature from temperate climates has reported occasional summer peaks for this pathogen, potentially linked to higher environmental bacterial loads in warmer conditions (39). The opportunistic nature of BRD-associated bacteria is further supported by findings from Cengiz et al. (49), who detected *P. multocida, M. haemolytica, H. somni,* and *M. bovis* using PCR in macroscopically healthy cattle lungs. Their study reported co-infections in several animals and emphasized the importance of these pathogens even in the absence of clinical signs or gross lesions. These findings align with our observations and reinforce the multifactorial and polymicrobial character of BRD, especially under subclinical or latent conditions that may go unnoticed without sensitive diagnostic methods.

These seasonal patterns are further complicated by inter-bacterial associations, such as the significant relationship observed between *T. pyogenes* and *Mycoplasma* spp. (*p* < 0.0001). Co-infections like these are particularly relevant during winter, when stress-induced immune suppression exacerbates disease severity. The synergistic mechanisms of these pathogens, such as enhanced tissue invasion or immune evasion, underscore the importance of understanding microbial interactions in BRD pathogenesis. The findings of this study emphasize the need for integrated management strategies to mitigate the compounded risks posed by farm size, seasonality, and pathogen dynamics. Tailored interventions, such as improving ventilation, reducing stocking density, and implementing strategic vaccination programs, are essential to control BRD in high-risk environments. These measures should be complemented by region-specific diagnostic approaches to account for the variability in pathogen prevalence and environmental conditions observed across different production systems.

The geographical variability in the prevalence of *Mycoplasma bovis*, *Trueperella pyogenes*, and *Mannheimia haemolytica* observed in our study aligns with evidence suggesting that management practices, climatic conditions, and herd densities contribute significantly to regional disparities in BRD prevalence. Our findings indicated that the central region of Spain showed the highest prevalence of *Mycoplasma* spp. (*p* < 0.0001), likely due to the interplay of intensive farm practices, higher animal density, and biosecurity challenges in this area. These results resonate with previous research highlighting the role of farm infrastructure and stocking densities as critical drivers of pathogen transmission (15). Nevertheless, a limitation of the present study is the absence of antimicrobial susceptibility testing, which prevents direct assessment of resistance patterns in the recovered isolates. Nonetheless, recent studies conducted in Spain and France have documented the presence of antimicrobial resistance genes in *Mycoplasma bovis*, highlighting concerning levels of resistance to macrolides, fluoroquinolones, and tetracyclines (50). These findings underline the need for ongoing AMR surveillance to support effective treatment strategies in the context of BRD.

Comparative studies offer valuable context for interpreting these findings. For instance, research in Australian feedlot cattle demonstrated that environmental stressors, including heat stress and poorly designed management systems, can exacerbate the colonization and pathogenicity of *Histophilus somni* and other BRD-associated pathogens (40). While these dynamics may differ in Spain due to climatic variability, they underscore the importance of adapting management practices to local conditions. Similarly, in Southern Brazil, the high prevalence of *M. bovis* in dairy herds has been attributed to frequent inter-farm animal movements and inconsistent biosecurity levels (41). This highlights the importance of controlling cross-regional transport and ensuring strict biosecurity protocols, which could mitigate similar risks in the Spanish context.

Furthermore, differences in antimicrobial resistance profiles and genomic variations among regions pose additional challenges for BRD control. Studies on *H. somni* have shown that regional genetic variability can influence antimicrobial susceptibility, complicating treatment strategies (40). In Spain, recent research identified two predominant molecular subtypes (ST2 and ST3) of *Mycoplasma bovis*, characterized by distinct antimicrobial resistance patterns, particularly toward fluoroquinolones (51). ST3 isolates displayed widespread fluoroquinolone resistance linked to specific mutations in the quinolone resistance-determining regions (QRDR) of gyrA and parC genes, reflecting selective pressures likely driven by extensive antimicrobial use in Spanish farms (51). These findings underscore the necessity of integrating genetic and antimicrobial resistance surveillance into regional BRD management frameworks to guide more precise therapeutic decisions. Future research should prioritize the identification of region-specific resistance patterns, particularly in pathogens like *Mycoplasma bovis* and *Trueperella pyogenes*, to inform evidence-based interventions effectively.

The absence of *Histophilus somni* isolates in our study, despite its documented role in other geographic regions (14), warrants further investigation. In particular, the low detection rate of *H. somni* in our study could be influenced by Spain’s regional climatic factors, which may be less conducive to its survival and transmission compared to other BRD-associated bacteria. Cooler, more humid conditions in regions like North America or Australia have been shown to favor *H. somni* prevalence, as reported in studies correlating environmental parameters with pathogen distribution (8). However, recent data from Spain has demonstrated the presence of *H. somni* at a prevalence of 24.1% across cattle farms, with the highest rates observed in fattening calves (27.8%) compared to adult cattle (12.1%) (42). Interestingly, studies on nasal shedding of *H. somni* indicate a wide range of prevalence rates depending on the detection method used. Moore et al. (43) found that nasal swabs showed a prevalence ranging from 0 to 9% when using culture methods, but when PCR-based detection was employed, the prevalence was significantly higher, reaching up to 42% in some populations. This suggests that *H. somni* may be present at subclinical levels or as a commensal organism in the upper respiratory tract, and its role as a primary pathogen may depend on additional predisposing factors such as co-infections or immune suppression. In the same study, *M. bovis*, *M. haemolytica*, and *P. multocida* were detected at 4.8, 13.4, and 26%, respectively, highlighting the variable presence of respiratory pathogens across different regions and management systems (43). Notably, the authors also reported that nasal isolation of *H. somni* at feedlot entry was not consistently associated with the development of clinical respiratory disease, indicating that detection alone may not be a reliable predictor of BRD onset. This discrepancy highlights the role of production systems and age groups in shaping the pathogen’s epidemiology. Notably, qPCR-based studies have consistently shown higher detection rates for *H. somni* compared to traditional culture methods, where sensitivity limitations often result in underdiagnosis (42, 52). In this context, the failure to detect *H. somni* in our study could be partially attributed to methodological constraints, as qPCR offers a clear advantage in identifying fastidious bacteria like *H. somni*. This underlines the importance of employing molecular diagnostic techniques to capture the true prevalence and role of this pathogen in BRD outbreaks. Alternatively, regional genetic variability among *H. somni* strains could explain its reduced prevalence in Spanish dairy systems. Incorporating advanced diagnostic tools, such as next-generation sequencing and targeted serological surveys, could provide greater clarity on the regional epidemiology of this pathogen. Such tools could also help identify cryptic infections or low-abundance strains that may not be detected by conventional culture methods. Using these advanced methodologies, future research could provide a clearer understanding of *H. somni* dynamics, helping to accurately define its role in BRD.

Overall, this study provides valuable insights into the epidemiology of BRD-associated bacterial pathogens in dairy heifers, highlighting key factors such as farm size, seasonality, and geographical region. These findings emphasize the importance of tailoring management and diagnostic approaches to address the specific risks posed by regional and environmental conditions. For instance, integrating PCR diagnostics into routine surveillance could enhance the detection of key pathogens like *M. bovis*, reducing the risk of underdiagnosis and enabling earlier interventions. These measures should also aim to reduce the reliance on antibiotics, particularly in intensive production systems where *M. bovis* is a major concern. While *M. bovis* itself is not zoonotic, its role as a driver of antimicrobial use raises concerns about the emergence and dissemination of resistance genes within bacterial populations (44–46). This highlights the importance of prudent antimicrobial stewardship to limit the broader impacts of resistance, which could eventually affect pathogens with zoonotic potential (47). Integrating preventative strategies, such as vaccination and improved biosecurity, with targeted antibiotic usage is critical for sustainable BRD management and mitigating public health risks. While the absence of *Histophilus somni* in our dataset raises questions about its epidemiological role in Spain, it underscores the need for further investigation using advanced diagnostic tools. Despite the robust dataset, some limitations, such as the potential underdiagnosis of pathogens and the exclusion of viral co-infections, warrant careful interpretation of the results. Another limitation of this study is that serological testing was limited to *M. bovis*. Although serological assays are available for other respiratory pathogens, including *M. haemolytica*, *P. multocida*, and *H. somni, M. bovis* was selected based on its critical role in chronic respiratory disease and its recognized epidemiological importance in Spanish dairy herds. Furthermore, logistical and resource constraints influenced the decision to focus serological efforts on this pathogen. Future studies incorporating broader serological panels would provide a more comprehensive understanding of co-infection dynamics and herd-level exposure to multiple BRD agents. In addition, future research should also focus on integrating genomic data, environmental factors, and molecular diagnostics to better understand pathogen dynamics in BRD. For example, next-generation sequencing could identify cryptic infections or low-abundance strains that may not be detected by traditional methods, offering a more comprehensive picture of pathogen prevalence and interactions. Addressing these gaps will support evidence-based interventions and improve the sustainability of dairy production systems.

## Data Availability

The raw data supporting the conclusions of this article will be made available by the authors, without undue reservation.
